# Storage Stability and Lipidomic Analysis Reveal the Effect of Frozen Storage Temperature on Pacific Saury (*Cololabis saira*)

**DOI:** 10.3390/foods14050756

**Published:** 2025-02-23

**Authors:** Ling Zhao, Shanyu Wang, Qi Liu, Rong Cao, Yating Zhang, Dong Su, Yueqin Yu

**Affiliations:** 1 State Key Laboratory of Eco-Chemical Engineering, College of Chemistry and Molecular Engineering, Qingdao University of Science and Technology, Qingdao 266042, China; zhaoling@ysfri.ac.cn (L.Z.); stella95730@163.com (Y.Z.); ziyousweet@163.com (D.S.); 2 Yellow Sea Fisheries Research Institute, Chinese Academy of Fishery Sciences, Qingdao 266071, China; wsy8587@foxmail.com (S.W.); liuqi@ysfri.ac.cn (Q.L.); caorong@ysfri.ac.cn (R.C.)

**Keywords:** *Cololabis saira*, oxidation, frozen storage temperature, lipidomics

## Abstract

Objectives: This study aimed to assess the effects of storage temperature on the lipidomics profile change in Pacific saury (*Cololabis saira*). Methods: In this paper, *C. saira* underwent frozen storage at two different temperatures, T1 (−18 °C) and T2 (−25 °C), for a duration of three months. Chemical and lipidomic methods were used to determine the changes in lipids during the storage process. Results: Results showed that the content of triglyceride and phospholipid decreased significantly (*p* < 0.05), and free fatty acid increased significantly (*p* < 0.05), while the content of total cholesterol remained relatively constant across different storage temperatures. Additionally, an increasing trend in AV, POV, and TBARS contents was observed after the freezing process, with lipid oxidation being significantly higher in the −18 °C group compared to the −25 °C group (*p* < 0.05). A comprehensive analysis identified 4854 lipid molecules in the muscles of *C. saira*, categorized into 46 lipid subclasses, predominantly including triglycerides (TG), phosphatidylcholine (PC), phosphatidylethanolamine (PE), phosphatidylinositol (PI), phosphatidylglycerol (PG), and diglycerides (DG). Among them, TG was the most abundant lipid, followed by PC. Using orthogonal partial least squares discriminant analysis (OPLS-DA) with a variable importance in projection (VIP) score > 1 and *p* value < 0.05 as criteria, 338, 271, and 103 highly significantly differentiated lipids were detected in the comparison groups CK vs. T1, CK vs. T2, and T1 vs. T2, respectively. The results indicated that storage at −18 °C had a more pronounced effect than storage at −25 °C. During the freezing process, TG expression was significantly down-regulated, and TG(18:4_14:0_20:5), TG(20:5_13:0_22:6), TG(22:6_14:1_22:6), and TG(18:4_13:0_22:6) were the most predominant individuals. The CK group was initially present in *C. saira* before storage. Differential lipid molecules in the CK vs. T1 and CK vs. T2 groups were screened using a fold change (FC) > 2 or FC < 0.5. In the CK vs. T2 group, 102 highly significant differential lipid molecules were identified, with 55 being down-regulated across seven subclasses. In contrast, the CK vs. T1 group revealed 254 highly significant differential lipid molecules, with 85 down-regulated across 13 subclasses. The results showed that more PCs and PEs were down-regulated, with a higher differential abundance of PE and PC in the −25 °C group compared to the −18 °C group. The differential metabolites were primarily enriched in 17 metabolic pathways, with glycerophospholipid metabolism being the most prominent, followed by sphingolipid metabolism during the frozen storage. Conclusions: Overall, −25 °C storage in production was more favorable for maintaining the lipid stability of *C. saira*. This work could provide useful information for aquatic product processing and lipidomics.

## 1. Introduction

*Cololabis saira* is a significant pelagic fish, which is widely distributed in the subtropical and temperate waters of the Pacific Ocean along the coasts of Asia and America [1]. In 2022, China’s commercial harvest of *C. saira* reached 35,477 tons [2]. *C. saira* is rich in protein, lipids, and other essential nutrients, and is particularly abundant in omega-3 polyunsaturated fatty acids [3,4,5]. The origin of *C. saira* is far from inland China, and it needs to undergo a long period of freezing and transport. Currently, sales of *C. saira* primarily consists of primary processed products such as frozen items, with fewer deep-processed products available, including convenient ready-to-eat pieces, canned, and boneless *C. saira*.

Freezing is one of the most widely used methods for preserving marine foods [6]. Currently, the standard storage temperature for most aquatic products in China is set at −18 °C. However, this temperature has not been optimal for lipid-rich aquatic products. For instance, the ideal freezing temperature for Atlantic salmon ranges between −45 °C and −60 °C [7]. Similarly, tuna requires storage and transportation at a minimum of −55 °C [8]. These specific temperature requirements are essential to slow down the deterioration in quality.

Although it partially inhibits most of the processes leading to quality deterioration, it does not completely prevent lipid oxidation and unfavorable changes in lipid quality and sensory properties [9]. A series of changes have occurred in fish meat during storage and processing, such as protein denaturation and lipid oxidation [10,11]. Many studies on *C. saira* have focused on lipid and flavor changes during processing [10,12,13] and the effects of natural antioxidants on lipid oxidation [4,12], but little work has been conducted on lipid oxidation of *C. saira* at different freezing temperatures; that is, apart from Tanaka et al. [14], who have investigated the quality changes of *C. saira* under different temperature storage conditions. However, comprehensive lipidomic profile differences due to the storage process had not been analyzed until now. Temperatures of −18 °C and −25 °C are commonly used in actual production. In this study, an untargeted lipidomic strategy was used to investigate the effect of storage temperature on lipid composition and lipid molecules during a −18 °C and −25 °C freezing storage process over three months. We screened the different lipids during storage at different temperatures and explored their metabolic pathways by detecting the dynamic changes in lipid profiles. This study aims to enhance the understanding of lipid profile changes during different frozen storage conditions and provide data support for the actual production of *C. saira*. Additionally, it offers a theoretical basis for the quality control of pelagic fishery catches.

## 2. Materials and Methods

### 2.1. Materials

A quantity of 180 kg of *Cololabis saira* was purchased from Penglai Jinglu Fisheries Co., Ltd. (Penglai, China) for this study. It was harvested from the North Pacific Ocean and initially frozen at −35 °C on the fishing vessel. The transportation from the North Pacific Ocean to China typically needed two or three months, during which the temperature was maintained at −18 °C. Upon arrival at Yantai port, *C. saira* were promptly transferred to the laboratory for further processing. The initial *C. saira* was labeled as CK, and the following fish were placed at −18 °C (labeled as T1) and −25 °C (labeled as T2) for frozen storage, respectively. In total, 60 kilogram fish were placed in storage for each group.

### 2.2. Reagents

Chloroform, methanol, isopropanol, sodium chloride, trichloroacetic acid (TCA), 95% ethanol, sodium hydroxide, sodium thiosulfate, acetic acid, starch indicator, potassium iodide, Triton X100, petroleum ether, thiobarbituric acid (TBA), toluene, copper acetate pyridine, ammonium formate, concentrated nitric acid, perchloric acid, and anhydrous sodium sulfate were analytically pure (Sinopharm Chemical Reagent Co., Ltd., Shanghai, China). MS-grade methanol, MS-grade acetonitrile, and HPLC-grade 2-propanol were purchased from Thermo Fisher Scientific Inc. (Waltham, MA, USA). HPLC-grade formic acid and HPLC-grade ammonium formate were purchased from Sigma Chemical Co. (St. Louis, MO, USA).

### 2.3. Lipid Composition and Lipid Oxidation Assay

The lipid composition of *C. saira* was analyzed at monthly intervals over a three-month period. Referring to the method of Wang et al. [15], 0.1 to 0.2 g of the total lipid sample was added to 20 mL digestive solution for wet digestion, the volume ratio of which (concentrated nitric acid and perchloric acid) was 4:1. Digestion continued until the digestive solution was essentially colorless. The digestion was fixed to 50 mL, and the phospholipid (PL) content in the total lipids was determined by the molybdenum blue colorimetric method. In total, 100 µL total lipid sample was diluted to the appropriate concentration. The diluted solution was made of isopropanol and TritonX100; its volume mass ratio was 9:1. Triglyceride (TG) and total cholesterol (TC) content were determined by following the kit instructions. Amounts of 5 mL of toluene and 1 mL of copper reagent (pH 6.1) were added to 0.10 g of the total lipid sample, and shaken for 2 min. Centrifugation was performed at 1000× *g* for 5 min, and the supernatant was taken to determine the Optical Density (OD) value at 715 nm. The standard curve was plotted with oleic acid to calculate the free fatty acid (FFA) content in the *C. saira* samples. Lipid oxidation of *C. saira* samples were assessed by measuring the acid value (AV), peroxide value (POV), and thiobarbituric acid reactive substances (TBARSs). AV was determined according to a thermal ethanol method of the Chinese standard GB 5009.229-2016 [16]. POV was determined by a titrimetric method of the Chinese standard GB 5009.227-2016 [17]. TBARSs were determined according to the spectrophotometric method of the Chinese standard GB 5009.181-2016 [18]. The detailed procedures of the assay methods can be found in the Supplementary Materials.

### 2.4. Determination of Lipid Profile

#### 2.4.1. Lipid Extraction

The lipidomic analysis was conducted at the end of the storage period (the third month), and the tissue of *C. saira* was rapidly frozen using liquid nitrogen and subsequently stored at −80 °C. Lipids of *C. saira* were extracted according to the Methyl tert-Butyl Ether (MTBE) method [19]. Briefly, a 200 µL volume of water was added to the sample, and then the sample was vortexed for 5 s. Subsequently, 240 µL of precooling methanol was added and the mixture was vortexed for 30 s. After that, 800 µL MTBE was added and the mixture was ultrasonicated for 20 min at 4 °C followed by sitting still for 30 min at room temperature. The solution was centrifuged at 14,000× *g* for 15 min at 10 °C and the upper organic solvent layer was obtained and dried under nitrogen.

#### 2.4.2. LC-MS/MS Method for Lipid Analysis

Reverse-phase chromatography was selected for LC separation using a CSH C18 column (1.7 µm, 2.1 mm × 100 mm, Waters Corporation, Milford, MA, USA). The lipid extracts were re-dissolved in 200 µL 90% isopropanol/acetonitrile, centrifuged at 14,000× *g* for 15 min, and, finally, 3 µL of sample was injected. Solvent A was acetonitrile–water (6:4, *v*/*v*) with 0.1% formic acid and 0.1 mM ammonium formate and solvent B was acetonitrile–isopropanol (1:9, *v*/*v*) with 0.1% formic acid and 0.1 mM ammonium formate. The initial mobile phase was 30% solvent B at a flow rate of 300 μL/min. It was held for 2 min, and then linearly increased to 100% solvent B in 23 min, followed by equilibrating at 5% solvent B for 10 min.

Mass spectra were acquired by Thermo Fisher Q-Exactive Plus in positive and negative modes. ESI parameters were optimized and preset for all measurements as follows: source temperature, 300 °C; capillary temp, 350 °C; the ion spray voltage for positive and negative ions was set at 3000 V; the S-Lens RF Level was set at 50%; and the scan range of the instruments was set at *m*/*z* 200–1800.

#### 2.4.3. Identification by Lipid Search

Lipid Search software version 4.2 (Thermo Scientific™) was used to identify the lipid molecules and lipid species based on MS/MS calculations [20]. It contains more than 30 lipid classes and more than 1,500,000 ion fragments in the database. Adducts of +H and +NH_4_ were selected for positive mode searches, and −H and +CH_3_COO were selected for negative mode searches since ammonium acetate was used in the mobile phases. Both mass tolerances for the precursor and fragment were set to 5 ppm, with a product ion threshold of 5%.

### 2.5. Statistical Analysis

The experimental data for lipid composition and oxidation were analyzed using SPSS 22.0 software, and significant differences were analyzed with Duncan’s multiple range test at *p* < 0.05. All lipidomic measurements were conducted in eight replicates. Regarding the data extracted from Lipid Search, lipids with a relative standard deviation (RSD) greater than 30% and missing values greater than 50% in each group were removed. After normalization and integration using the Perato scaling method, the processed data were imported into SIMPCA-P 16.0 (Umetrics, Umea, Sweden) for principal component analysis (PCA).

## 3. Results

### 3.1. Lipid Composition Change and Lipid Oxidation Analysis of C. saira at Different Storage Temperatures

**Figure 1:**
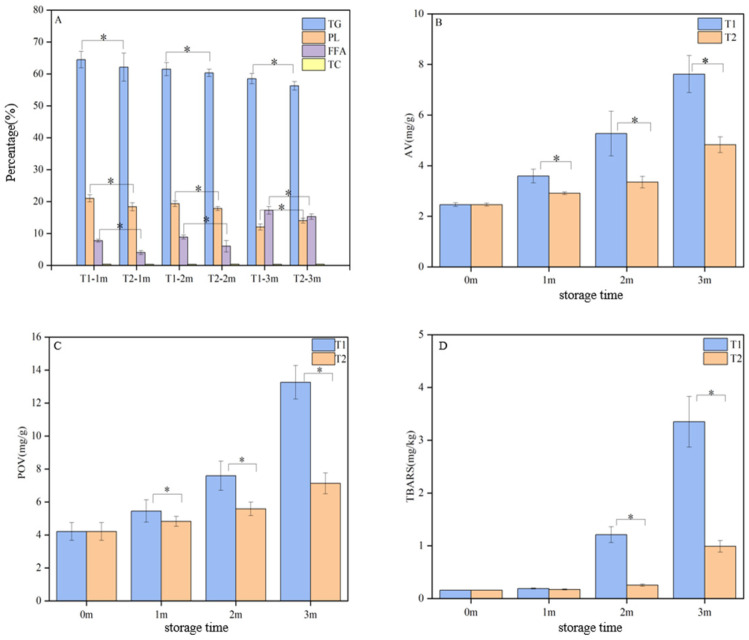
Lipid composition (**A**) and lipid oxidation (**B**–**D**) analysis of *C. saira* at different storage temperatures; asterisks indicate statistical significance (* *p* < 0.05).

Figure 1A illustrates the lipid composition of *C. saira* at various storage temperatures, providing insights into the evolution of lipids during the frozen storage process. Similarly to most marine fish, the lipid composition of *C. saira* was predominantly comprised of TG and PL, with smaller quantities of FFA and very low levels of TC. During storage, the contents of TG and PL decreased significantly (*p* < 0.05), while FFA levels increased significantly (*p* < 0.05). However, the TC content remained relatively constant at different storage temperatures. Notably, the changes in lipid composition were more pronounced in the T1 group (−18 °C) compared to the T2 group (−25 °C), suggesting that storage temperature significantly influences lipid composition. Consequently, a storage temperature of −25 °C was more suitable for the long-term frozen storage of *C. saira*.

To assess the levels of primary and secondary oxidation products during the lipid oxidation process, AV, POV, and TBARS were utilized as key indicators [21]. Figure 1 illustrates the lipid oxidation of *C. saira* at various frozen storage temperatures. It is evident that lipid oxidation was typically unavoidable during frozen storage, leading to significant increases in the AV, POV, and TBARS values of *C. saira*.

Figure 1B illustrates the changes in the AV of *C. saira* at various storage temperatures, revealing a gradual increase in AV with the extension of the storage period. Notably, the AV in the T1 group increased significantly more than in the T2 group (*p* < 0.05). This observation aligned with the results from the lipid composition analysis, suggesting a potential link to the hydrolysis of TC and PL. Furthermore, the rate of lipid hydrolysis was influenced by several factors, including the moisture content and its state within the samples, as well as the activity of endogenous lipase and other variables [22,23]. POV was an index used to determine the content of primary oxidation products, such as hydroperoxides, which can reflect the degree of the primary oxidation of lipids [24]. Aquatic products, such as *C. saira*, are rich in unsaturated fatty acids, which were prone to oxidative decomposition during storage, leading to the formation of malondialdehyde. The TBARS assay was utilized to evaluate lipid secondary oxidation by measuring the malondialdehyde content produced during this process [25]. Lipid oxidation was generally unavoidable during transportation or storage, resulting in significant increases in both POV and TBARS values in *C. saira*. Figure 1C,D illustrate the changes in POV and TBARSs, respectively, at different storage temperatures. The POV and TBARS values of the T1 group increased significantly compared to the T2 group (*p* < 0.05).

Overall, an increasing trend in AV, POV, and TBARS contents was observed during the freezing process. These results indicated that storage at −25 °C is more conducive to the preservation of *C. saira*.

### 3.2. Lipidomics Analysis of C. saira at Different Storage Temperatures

During the storage process, lipid oxidation can be influenced by various factors, including temperature, duration, and packaging. When lipid peroxidation occurs, the appearance of *C. saira* may become tawny, and it may develop an unpleasant odor or a hard texture, which negatively impacts consumer sensory acceptability. Therefore, understanding the fundamental processes and mechanisms of lipid oxidation is crucial for ensuring the storage stability of *C. saira*.

Table 1 presented the statistical results obtained from both positive and negative ion modes. In the muscles of *Cololabis saira,* a total of 4854 lipid molecules were identified and categorized into 46 lipid subclasses. These subclasses primarily included triglycerides (TG), phosphatidylcholine (PC), phosphatidylethanolamine (PE), phosphatidylinositol (PI), phosphatidylglycerol (PG), and diglycerides (DG). Notably, TG was the most abundant lipid in *C. saira*, serving as the primary storage form of fatty acids in organisms, as well as an essential energy source and carrier [26]. PC was the second most prevalent lipid, recognized as a major component of fish phospholipids, and it plays a critical role in maintaining cell membrane permeability and structural integrity [27].

### 3.3. Multivariate Statistical Analysis of C. saira at Different Storage Temperatures

**Figure 2:**
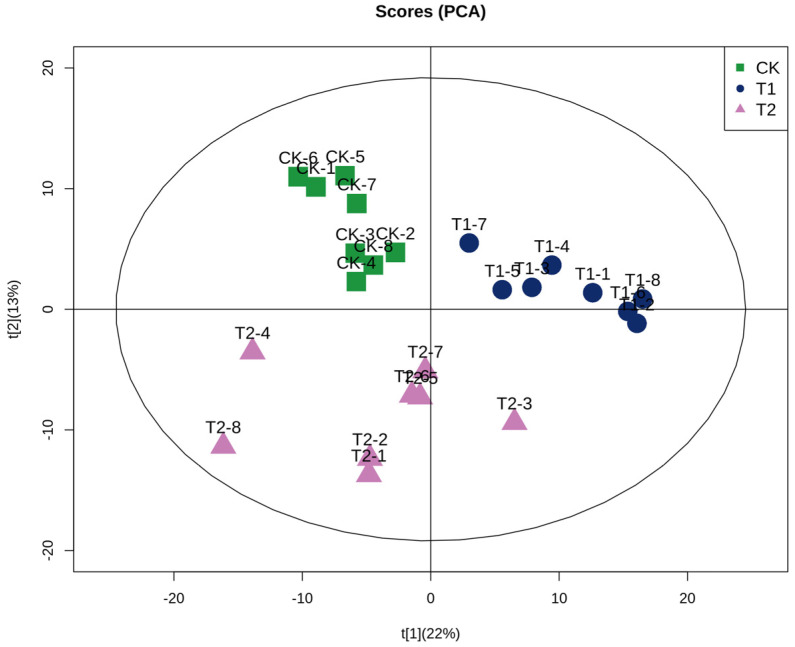
Principal component analysis.

In order to elucidate the changes in the lipid profile of *C. saira* under different storage temperatures and identify potential differences in lipid types, a principal component analysis (PCA) model was established, as illustrated in Figure 2. The figure demonstrated the distribution of the lipid profiles in the three groups of *C. saira*, from which it could be seen that the distribution of the same group of *C. saira* was concentrated in a region. The samples of TI and T2 groups were dispersed in different regions and far away from each other, which indicated that there were significant differences in the lipid profiles of the T1 and T2 group of *C. saira*, and further confirmed that storage temperature has a substantial effect on the lipid profiles of *C. saira*.

### 3.4. Differentially Abundant Lipid Analysis

**Figure 3:**
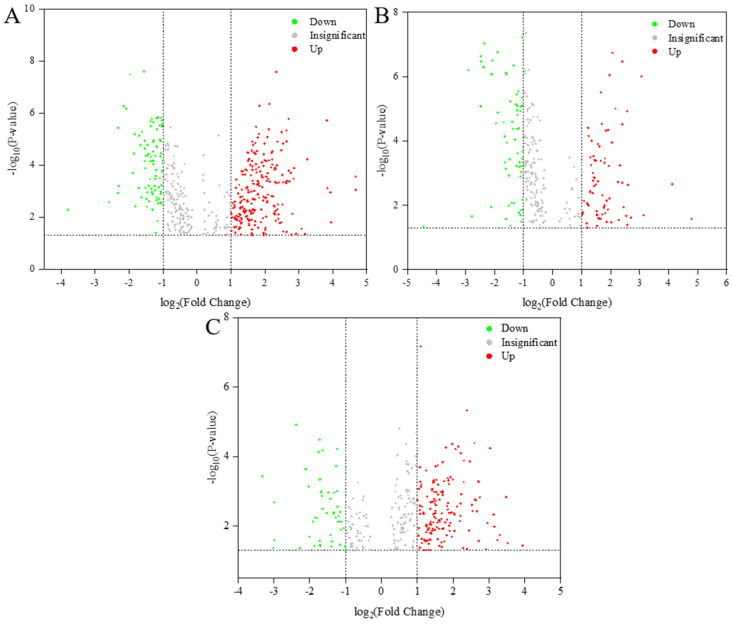
Volcano plot of differential lipid molecules in CK versus T1 sample (**A**), CK versus T2 sample (**B**), T1 versus T2 sample (**C**).

In lipidomics studies, a high Variable Importance in Projection (VIP) score combined with a low *p*-value has indicated significant differences in target lipid samples [28]. By employing Orthogonal Partial Least Squares Discriminant Analysis (OPLS-DA) with VIP > 1 and *p*-value < 0.05 as the criteria, we identified highly significantly differentiated lipid molecules (Figure 3). Specifically, 338, 271, and 103 highly significantly differentiated lipids were detected in the comparison groups CK vs. T1, CK vs. T2, and T1 vs. T2, respectively. Figure 3 presented volcano plots of the three comparison groups, illustrating the changes in lipid compounds. In these plots, each point represents a lipid compound, with larger values along the horizontal and vertical axes indicating more significant alterations in the selected lipid compounds. The figure reveals that the relative contents of most lipid metabolites were either significantly up-regulated or down-regulated across the three sample groups. These findings suggested that the most pronounced differences in the lipid composition of *C. saira* occurred during T1 storage. The results indicated that the T1 group storage had a more substantial impact on lipid quality compared to the T2 group.

The lipid molecules that were either up-regulated or down-regulated in the CK vs. T1 and CK vs. T2 groups were identified using a fold change (FC) criterion of >2 or <0.5. The analysis revealed that the down-regulation of triglycerides (TG) was the main reason for the changes in the lipid profiles of *C. saira* during storage, and thus the lipid compounds that were down-regulated lipid molecules in the different comparison groups were listed in Table 2 and Table 3, separately. In the CK vs. T2 group, 102 highly significant differential lipid molecules were detected, with 55 being down-regulated. These down-regulated molecules belonged to seven subclasses, including 40 species of TG, 4 species of sphingomyelins (SM), 3 species of sterols (ST), 3 species of sphingolipids (SPH), 3 species of PI, 1 species of PG, and 1 species of phosphatidylserine (PS). In comparison, the CK vs. T1 group exhibited 254 highly significant differential lipid molecules, with 85 being down-regulated across 13 subclasses. These included 33 species of TG, 17 species of PE, 11 species of phosphatidylcholines (PC), 5 species of PI, 5 species of ST, 4 species of PG, 2 species of SPH, 2 species of SM, 2 species of cardiolipins (CL), 1 species of LPE, 1 species of Dihexosylceramide (Hex2Cer), 1 species of DG, and 1 species of Phosphatidylserine (PS). This suggested a more complex lipid metabolism in this reservoir. These lipid compounds could also serve as differentiating factors. The findings indicated that the lipid profiles of *C. saira* were significantly influenced by different storage temperatures, aligning with previous studies on lipid content and oxidation.

**Figure 4:**
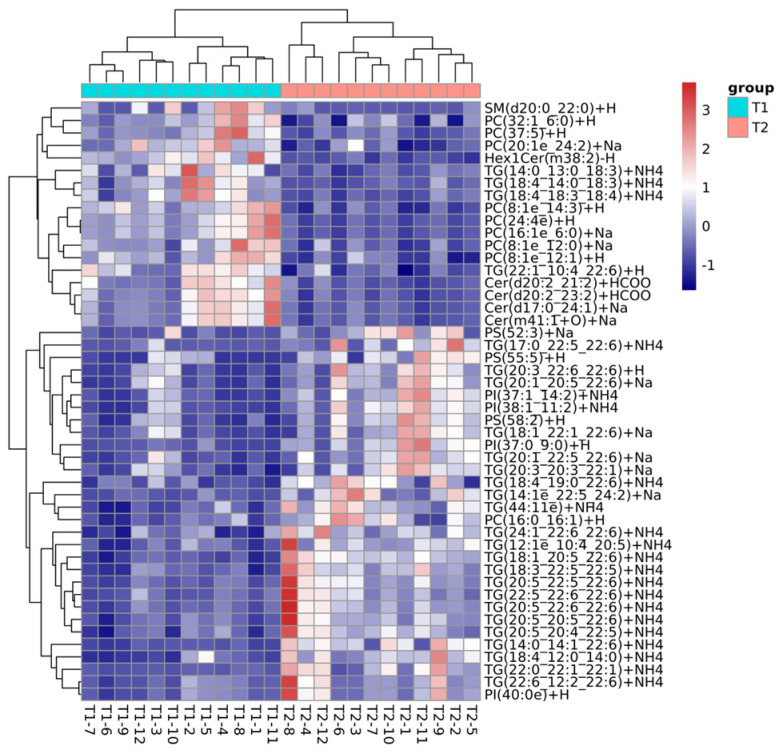
The correlation analysis between the lipid metabolites and different frozen storage temperature.

During the freezing storage process, the expression of TG was significantly down-regulated (Figure 4), primarily decomposing into FFA, DG, and monoglycerides (MG). These metabolites served as energy sources and substrates for the proliferation of cryophilic microorganisms, facilitating microbial growth [26]. TG(18:4_14:0_20:5), TG(20:5_13:0_22:6), TG(22:6_14:1_22:6), and TG(18:4_13:0_22:6) were the most predominant species. The freezing storage process also led to an increase in AV, POV, and TBARSs, indicating a comparative deterioration of the lipid profiles in *C. saira*. This reduction in TG levels can be attributed to oxidative degradation, which is triggered by hydrolysis due to the enzymatic action of lipases [28]. This process might convert TG into substances such as DG, while simultaneously releasing fatty acids [29]. DG, an intermediate product of lipid metabolism, is closely associated with the biosynthesis and catabolism of TG [30]. Notably, the T1 and T2 groups exhibited two up-regulated DG lipid species. PE and PC played crucial roles in regulating energy, lipid metabolism, and lipoprotein secretion [31]. The expression of functional ingredients such as PE, PC, and PI was also significantly down-regulated, suggesting their conversion to lysophosphatidylethanolamine (LPE), lysophosphatidylcholine (LPC), and lysophosphatidylinositol (LPI), respectively, through pathways such as partial hydrolysis or lipid oxidation [27]. These findings underscored the critical role of lipid oxidation in lipid transformations during the freezing storage process of *C. saira*, in accordance with the results of previous studies [22,32].

Comprehensive analysis of the differential lipids at freezing temperatures of −18 °C and −25 °C revealed significant findings regarding lipid regulation and oxidation in muscle tissue. The study found that more phosphatidylcholines (PCs) and phosphatidylethanolamines (PEs) exhibited down-regulation, likely due to oxidation reactions in muscle tissue. These reactions may cause hydrogen rearrangement on the PC/PE chains and lead to the breakage of the C-C layer of α-bonds [33]. Notably, the differential abundance of PE and PC was higher in the −25 °C group compared to the −18 °C group, indicating that a freezing temperature of −25 °C could more effectively inhibit lipid oxidation in the muscle of *C. saira*. These results underscored the significant impact of different freezing storage temperatures on the lipid profile, with −25 °C being more favorable for the frozen storage of *C. saira*. This finding aligned with previous results concerning lipid composition and oxidation.

### 3.5. Lipid Metabolic Pathway Analysis of C. saira During Storage Process

**Figure 5:**
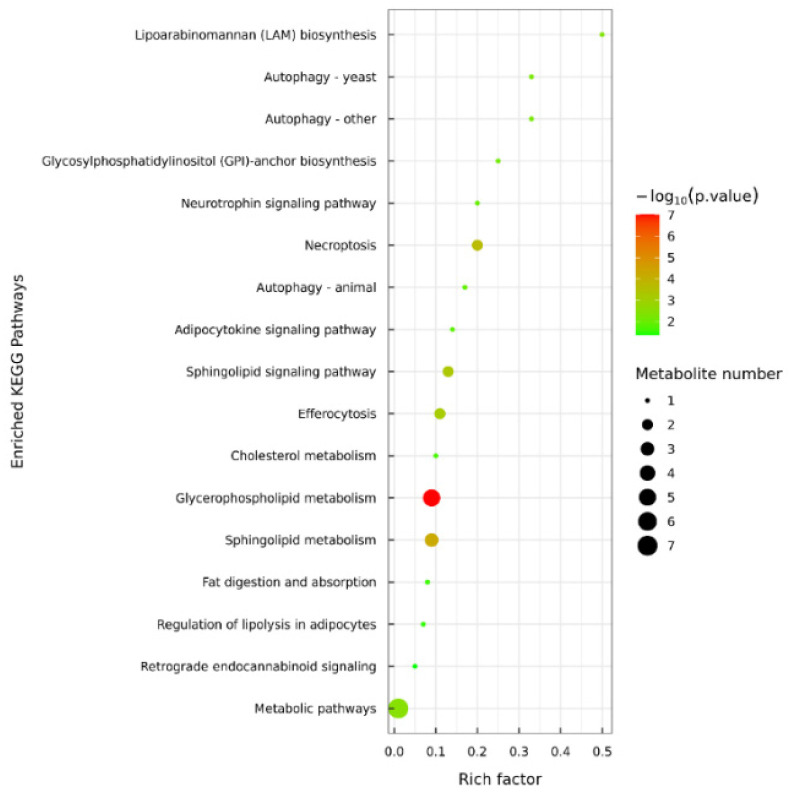
KEGG pathway analysis of highly significant differential lipids in T1 vs. T2 group.

The analysis of metabolic pathways provided insights into how frozen storage temperatures affect lipid distribution in samples, as indicated in previous studies [6]. Utilizing the KEGG database, the pathway annotation analysis of lipid differential metabolites in *C. saira* at varying storage temperatures was depicted in Figure 5. Comparative analysis revealed that these differential metabolites were predominantly enriched in 17 metabolic pathways of the T1 (−18 °C) vs. T2 (−25 °C) group. Among these, glycerophospholipid metabolism emerged as the primary pathway enriched during the frozen storage process. Key lipid components such as phosphatidylcholine (PC), phosphatidylethanolamine (PE), phosphatidylinositol (PI), and phosphatidylserine (PS) were annotated within this pathway. These lipids were crucial as they constitute major components of cell membranes, thereby maintaining cellular physiological functions and facilitating the transport of triglycerides [34]. Additionally, sphingolipid metabolism was identified as another significant pathway enriched during frozen storage, with ceramide (Cer) and sphingomyelin (SM) being the two significantly different lipid metabolites annotated in this pathway. Further investigation into the lipid oxidation pathway is necessary, in conjunction with metabolomics, to uncover the potential key lipid molecules involved in the storage process of *C. saira.*

## 4. Conclusions

In conclusion, the findings indicated that storage temperature had a significant influence on the lipid stability of *C. saira* during the frozen storage process. The lipid stability of *C. saira* stored at −18 °C was much worse than that at −25 °C. Thus, −25 °C storage was more conducive to maintain the storage stability of *C. saira* in production. The study will provide valuable insights into production quality control of pelagic fishery catches.

## Figures and Tables

**Table 1:** Number of lipid subclasses and compounds.

Lipid Subclass	Number of Compounds
Triglycerides, TG	1182
Phosphatidylcholine, PC	430
Phosphatidylethanolamine, PE	409
Phosphatidylinositol, PI	330
Phosphatidylglycerol, PG	327
Diglyceride, DG	316
Phosphatidylserine, PS	224
Cardiolipin, CL	205
Ceramide, Cer	127
Sulfatide, ST	108
Hexosylceramide, Hex1Cer	97
Sphingomyelin, SM	75
Dihexosylceramide, Hex2Cer	74
Lysophosphatidylcholine, LPC	58
Simple Glc series, Hex3Cer	51
N-acetylhexosyl ceramide, CerG2GNAc1	45
Phytosphingosine, phSM	44
Lysophosphatidylethanolamine, LPE	39
Phosphatidylinositol phosphate, PIP	29
Phosphatidylinositol bisphosphate, PIP2	29
Wax exters, WE	26
Aacyl carnitine, AcCa	22
Sphingosine bases, SPH	22
Monoglyceride, MG	21
Phosphatidic acid, PA	20
Ganglioside, monosialo trihexosyl ceramide, GM3	16
Lysophosphatidylglycerol, LPG	14
Ceramides phosphate, CerP	13
Fatty acid, FA	13
Lysophosphatidylinositol, LPI	10
Lysophosphatidylserine, LPS	9
Zymosterol ester, ZyE	8
(O-acyl)-1-hydroxy fatty acid, OAHFA	4
Cholesteryl esters, ChE	3
Coenzyme, Co	3
Phosphatidylinositol triphosphate, PIP3	3
Ganglioside, disialo tetrahexosyl ceramide, GD1a	2
Ganglioside, disialo dihexosyl ceramide, GD2	2
monosialotetrahexosyl ganglioside, GM1	2
Ganglioside, trisialo trihexosyl, GT3	2
Sphin-gosine phosphate, SPHP	2
Dihexosyl N-acetylhexosyl ceramide, CerG3GNAc1	1
Disialoganglioside, GD3	1
Lysophosphatidic acid, LPA	1
Sitosteryl ester, SiE	1
Stigmasterol ester, StE	1

**Table 2:** Changes in significantly different lipid molecules in CK vs. T1 group during storage process.

Number	Lipid lon	Class	Fold Change	*p*-Value	VIP
1	PE(18:1p_22:6)+H	PE	0.499098	6.44 × 10^−5^	1.042086
2	PC(39:6)+H	PC	0.496688	8.43 × 10^−5^	1.38837
3	TG(16:0_14:4_22:6)+NH_4_	TG	0.492149	2.84 × 10^−6^	1.881346
4	TG(20:5_13:0_22:6)+NH_4_	TG	0.488128	3.24 × 10^−6^	3.105524
5	TG(22:6_14:1_22:6)+NH_4_	TG	0.487257	0.000175	3.042199
6	PE(16:0_22:6)−H	PE	0.486444	1.94 × 10^−6^	2.852529
7	PE(35:2e)+Na	PE	0.48616	0.000594	1.108355
8	SM(d20:0_24:5)+H	SM	0.480833	0.00303	1.631861
9	CL(78:11)−2H	CL	0.480744	1.49 × 10^−6^	2.871019
10	TG(22:6_14:3_22:6)+NH_4_	TG	0.47991	0.002727	1.572998
11	PS(43:1e)+H	PS	0.478902	3.56 × 10^−5^	2.906576
12	PC(42:9)+H	PC	0.47835	0.001059	1.089234
13	TG(18:4_14:0_20:5)+NH_4_	TG	0.477786	0.001276	9.197348
14	PC(22:3_14:1)+H	PC	0.47614	1.19 × 10^−5^	1.162553
15	TG(18:4_14:0_18:4)+NH_4_	TG	0.475079	4.68 × 10^−5^	6.977423
16	PI(16:0_22:6)−H	PI	0.469722	0.000323	1.639228
17	PC(22:1_11:2)+Na	PC	0.467923	1.05 × 10^−5^	4.199848
18	PG(43:3e)+NH_4_	PG	0.466328	3.02 × 10^−6^	3.178235
19	PE(10:0e_6:0)+H	PE	0.463883	0.002008	1.500992
20	ST(d17:1_25:0)+NH_4_	ST	0.457943	0.000698	1.70078
21	PI(40:0e)+H	PI	0.457439	0.000708	1.704323
22	PC(17:1_22:6)+HCOO	PC	0.455686	1.43 × 10^−6^	1.153681
23	PE(16:0_18:2)+Na	PE	0.454466	0.003023	1.162741
24	TG(18:4_12:0_18:4)+NH_4_	TG	0.444249	0.001779	2.19355
25	TG(18:4_18:0_22:6)+H	TG	0.441096	4.30 × 10^−6^	1.808455
26	PE(18:1p_20:5)+H	PE	0.440797	2.07 × 10^−5^	1.528535
27	TG(18:4_18:4_22:6)+H	TG	0.439262	1.60 × 10^−6^	1.637022
28	PG(32:2e)+NH_4_	PG	0.436992	1.91 × 10^−5^	1.147128
29	TG(18:4_18:3_20:5)+NH_4_	TG	0.435734	0.000153	4.625362
30	LPE(16:0)−H	LPE	0.435246	0.002236	2.086989
31	TG(22:6_12:2_22:6)+NH_4_	TG	0.431817	0.000265	2.296603
32	PG(18:1e_23:1)+NH_4_	PG	0.427813	4.44 × 10^−5^	1.735106
33	TG(18:4_14:1_18:4)+NH_4_	TG	0.425433	0.000918	1.645381
34	PC(20:5_22:6)+HCOO	PC	0.424829	0.000338	1.648933
35	PC(44:11)+H	PC	0.417744	0.000639	1.998421
36	PC(22:6_22:6)+HCOO	PC	0.414333	1.13 × 10^−5^	2.111974
37	TG(18:4_13:0_22:6)+NH_4_	TG	0.411841	2.24 × 10^−6^	3.32173
38	TG(18:4_18:4_20:5)+NH_4_	TG	0.411485	6.69 × 10^−5^	5.206043
39	TG(18:4_18:3_18:4)+NH_4_	TG	0.411481	9.19 × 10^−5^	2.389019
40	TG(18:4_13:0_20:5)+NH_4_	TG	0.408598	1.28 × 10^−5^	1.884269
41	PE(18:2e_22:6)−H	PE	0.4084	2.98 × 10^−5^	1.0287
42	PE(18:1_22:6)−H	PE	0.404044	0.005235	1.597052
43	CL(82:13)−2H	CL	0.403207	0.005028	1.601206
44	TG(20:4_22:6_23:1)+NH_4_	TG	0.402977	0.001303	1.084609
45	ST(m42:0 + O)+NH_4_	ST	0.401593	7.04 × 10^−5^	4.169806
46	ST(d15:0_25:0)+NH_4_	ST	0.398087	0.001072	1.264458
47	PI(33:1)+NH_4_	PI	0.397697	1.66 × 10^−6^	1.082869
48	DG(21:5e)+NH_4_	DG	0.39523	0.000806	1.177416
49	PG(31:2e)+NH_4_	PG	0.390305	3.11 × 10^−6^	1.162939
50	TG(16:0_14:1_22:6)+H	TG	0.389657	4.88 × 10^−5^	1.350519
51	TG(18:4_12:0_20:5)+NH_4_	TG	0.387985	2.06 × 10^−6^	2.156707
52	TG(22:4_12:4_22:4)+NH_4_	TG	0.3853	7.13 × 10^−5^	2.344739
53	PC(20:5_22:6)+H	PC	0.383516	0.00035	1.10243
54	PC(17:1_22:6)+H	PC	0.375831	0.002524	2.368831
55	PC(37:5)+H	PC	0.372954	3.74 × 10^−5^	2.477881
56	TG(18:4_14:3_22:6)+NH_4_	TG	0.372685	0.000631	1.676399
57	PI(42:1e)+H	PI	0.364115	1.67 × 10^−5^	1.105667
58	SPH(m18:1)+H	SPH	0.363043	4.33 × 10^−5^	1.437811
59	PE(16:0_20:5)−H	PE	0.362863	0.001141	1.06783
60	PE(39:2)+Na	PE	0.359881	0.003976	1.106558
61	PE(29:1_11:3)+Na	PE	0.356409	0.00195	2.475733
62	TG(18:4_18:4_18:4)+NH_4_	TG	0.350723	4.43 × 10^−5^	3.849431
63	PE(20:1_22:6)−H	PE	0.348642	0.000763	1.674806
64	TG(18:4_14:2_20:5)+NH_4_	TG	0.348242	0.000135	1.611142
65	TG(11:0_18:4_22:6)+NH_4_	TG	0.344356	2.29 × 10^−5^	1.024028
66	PE(16:1e_22:6)−H	PE	0.343037	1.16 × 10^−5^	1.600446
67	TG(18:4_14:3_20:5)+NH_4_	TG	0.342678	7.13 × 10^−5^	1.382108
68	TG(22:1_10:4_22:6)+H	TG	0.339259	2.49 × 10^−8^	1.694093
69	ST(d17:1_24:0)+NH_4_	ST	0.324952	5.26 × 10^−6^	1.074646
70	TG(20:5_12:1_22:6)+H	TG	0.324425	0.001746	1.569025
71	SPH(d20:0)+H-H_2_O	SPH	0.314818	1.68 × 10^−5^	1.334997
72	ST(d48:3)+Na	ST	0.306939	7.41 × 10^−6^	1.166141
73	PE(18:2_22:6)+Na	PE	0.305299	0.000763	1.272167
74	TG(20:2_14:4_22:5)+NH_4_	TG	0.285599	0.003817	1.646126
75	PE(22:6_22:6)−H	PE	0.280681	6.49 × 10^−6^	2.91534
76	PE(20:3_22:6)+Na	PE	0.27609	3.58 × 10^−5^	3.615534
77	PE(20:5_22:6)−H	PE	0.271036	0.000203	1.163258
78	TG(18:4_20:5_22:6)+H	TG	0.255424	3.27 × 10^−8^	1.147957
79	TG(20:5_14:2_22:6)+H	TG	0.236534	6.83 × 10^−7^	1.309602
80	TG(18:1_11:3_21:0)+Na	TG	0.224134	5.49 × 10^−7^	1.208512
81	TG(18:4_18:4_20:5)+H	TG	0.202142	0.000627	2.141881
82	TG(22:6_11:2_21:0)+H	TG	0.201635	3.72 × 10^−6^	2.215741
83	Hex2Cer(m39:3)+H-H_2_O	Hex2Cer	0.199907	0.001191	1.061588
84	SM(d20:0_22:0)+H	SM	0.166036	0.002705	8.710905
85	PI(25:1_11:2)+NH_4_	PI	0.071681	0.005298	7.23351

**Table 3:** Changes in significantly different lipid molecules in CK vs. T2 group during storage process.

Number	Lipidlon	Class	Fold Change	*p*-Value	VIP
1	TG(20:5_22:1_22:6)+NH_4_	TG	0.49851	5.68 × 10^−7^	5.215054
2	TG(20:5_22:6_22:6)+NH_4_	TG	0.491599	0.000221	2.567459
3	TG(18:1_20:5_22:6)+NH_4_	TG	0.490677	6.05 × 10^−8^	4.438805
4	TG(20:0_22:3_23:0)+Na	TG	0.489055	7.87 × 10^−6^	4.994488
5	TG(24:1_22:1_24:1)+NH_4_	TG	0.486977	0.006783	1.564398
6	TG(22:6_14:1_22:6)+NH_4_	TG	0.486156	0.000355	2.537809
7	TG(18:4_16:0_20:5)+NH_4_	TG	0.482545	4.57 × 10^−6^	9.422562
8	TG(18:4_18:4_20:5)+NH_4_	TG	0.475521	0.000612	3.973146
9	TG(20:5_14:1_22:6)+NH_4_	TG	0.471436	8.49 × 10^−6^	1.658027
10	TG(22:4_12:4_22:4)+NH_4_	TG	0.470116	0.000904	1.769338
11	TG(26:1_22:1_22:6)+NH_4_	TG	0.467412	0.001272	1.034124
12	TG(20:5_18:2_22:6)+NH_4_	TG	0.460452	1.21 × 10^−5^	3.218636
13	TG(24:1_22:1_22:1)+NH_4_	TG	0.456162	0.000451	4.562797
14	ST(m42:0 + O)+NH4	ST	0.451775	0.000622	3.284288
15	TG(18:4_16:1_20:5)+NH_4_	TG	0.449565	3.97 × 10^−5^	4.416179
16	TG(20:5_20:5_22:6)+NH_4_	TG	0.440813	2.87 × 10^−6^	5.177796
17	TG(22:5_20:4_22:6)+NH_4_	TG	0.433843	0.000243	1.074286
18	TG(22:1_22:1_22:1)+NH_4_	TG	0.432794	8.21 × 10^−6^	9.731679
19	ST(d46:2)+NH_4_	ST	0.432073	8.33 × 10^−6^	5.279914
20	SM(d18:2_24:1)+H	SM	0.430173	4.26 × 10^−5^	1.308388
21	TG(18:4_20:5_22:6)+NH_4_	TG	0.429418	1.56 × 10^−5^	6.177515
22	TG(14:0_20:5_22:6)+NH_4_	TG	0.429232	1.12 × 10^−5^	2.991447
23	TG(20:1_11:3_22:6)+NH_4_	TG	0.426299	3.61 × 10^−6^	1.616654
24	TG(18:4_14:1_18:4)+NH_4_	TG	0.424032	0.000601	1.434173
25	TG(18:4_18:3_22:6)+NH_4_	TG	0.41912	7.34 × 10^−6^	1.093412
26	TG(16:0_14:1_22:6)+H	TG	0.406657	9.14 × 10^−5^	1.130789
27	TG(20:5_12:1_22:6)+H	TG	0.395436	0.008554	1.019339
28	TG(16:0_14:4_22:6)+NH_4_	TG	0.395024	4.52 × 10^−7^	1.802223
29	TG(18:4_14:3_16:0)+NH_4_	TG	0.389088	4.19 × 10^−5^	1.988512
30	PG(43:3e)+NH4	PG	0.380387	2.56 × 10^−5^	2.920494
31	PI(37:0_9:0)+H	PI	0.371176	0.008509	1.172954
32	SM(d20:0_24:3)+H	SM	0.36418	5.99 × 10^−6^	2.831523
33	SM(d20:0_22:2)+H	SM	0.35894	0.000375	2.3627
34	TG(22:6_11:2_21:0)+H	TG	0.355089	0.001193	1.531593
35	TG(18:4_12:0_18:4)+NH_4_	TG	0.338135	0.000114	2.230751
36	TG(16:1_14:0_20:5)+NH_4_	TG	0.331458	8.32 × 10^−7^	7.620491
37	TG(12:0_14:0_22:6)+NH_4_	TG	0.328791	0.000737	3.230222
38	TG(18:4_12:0_20:5)+NH_4_	TG	0.327852	7.66 × 10^−7^	1.988383
39	TG(18:4_12:0_14:0)+NH_4_	TG	0.31954	0.000463	1.849902
40	TG(18:4_14:0_20:5)+NH_4_	TG	0.318295	7.37 × 10^−5^	10.2416
41	TG(18:4_14:3_20:5)+NH_4_	TG	0.311052	2.61 × 10^−5^	1.263194
42	TG(18:4_14:2_20:5)+NH_4_	TG	0.270653	1.31 × 10^−5^	1.556192
43	TG(28:1_22:3_22:6)+NH_4_	TG	0.269109	1.76 × 10^−7^	1.040046
44	SM(d20:0_24:5)+H	SM	0.259478	2.83 × 10^−5^	1.932346
45	TG(18:4_13:0_22:6)+NH_4_	TG	0.237456	3.20 × 10^−7^	3.431831
46	PI(42:1e)+H	PI	0.233915	8.54 × 10^−7^	1.113729
47	PS(43:1e)+H	PS	0.232173	8.28 × 10^−7^	3.321953
48	SPH(m18:1)+H	SPH	0.195451	9.37 × 10^−8^	1.459295
49	ST(d17:1_25:0)+NH_4_	ST	0.19223	5.03 × 10^−7^	2.081955
50	PI(40:0e)+H	PI	0.191481	5.31 × 10^−7^	2.086004
51	ST(d15:0_25:0)+NH_4_	ST	0.18241	8.61 × 10^−6^	1.444491
52	SPH(d20:0)+H-H_2_O	SPH	0.181566	3.37 × 10^−7^	1.301676
53	TG(22:6_12:2_22:6)+NH_4_	TG	0.180922	2.34 × 10^−7^	2.711327
54	TG(18:4_14:3_22:6)+NH_4_	TG	0.176824	8.09 × 10^−6^	1.854093
55	TG(22:6_14:3_22:6)+NH_4_	TG	0.134366	6.24 × 10^−7^	2.144745

## Data Availability

The original contributions presented in the study are included in the article/Supplementary Materials, further inquiries can be directed to the corresponding author.

## Supplementary Materials

The following supporting information can be downloaded at https://www.mdpi.com/article/10.3390/foods14050756/s1, Determination of lipid oxidation.
