# Supplementary material for: Storage Stability and Lipidomic Analysis Reveal the Effect of Frozen Storage Temperature on Pacific Saury (Cololabis saira)
Source: Foods. 2025 Feb 23;14(5):756. doi: 10.3390/foods14050756 (PMC11898982; doi:10.3390/foods14050756)
Supplement: Supplementary file 1 [file foods-14-00756-s001.zip › foods-3425264-supplementary.pdf]

Supplementary Material

Storage Stability and Lipidomic Analysis Reveal the Effect of Frozen Storage Temperature on Pacific Saury (*Cololabis saira*)

Ling Zhao [1,2], Shanyu Wang [2], Qi Liu [2], Rong Cao [2], Yating Zhang [1], Dong Su [1] and Yueqin Yu [1,*]

[1] State Key Laboratory of Eco-Chemical Engineering, College of Chemistry and Molecular Engineering, Qingdao University of Science and Technology, Qingdao 266042, China; zhaoling@ysfri.ac.cn (L.Z.); stella95730@163.com (Y.Z.); ziyousweet@163.com (D.S.)
[2] Yellow Sea Fisheries Research Institute, Chinese Academy of Fishery Sciences, Qingdao 266071, China; wsy8587@foxmail.com (S.W.); liuqi@ysfri.ac.cn (Q.L.); caorong@ysfri.ac.cn (R.C.)
* Correspondence: qustyu@163.com

Additional Methods

1 Determination of AV

AV is determined according to thermal ethanol method of Chinese standard GB 5009.229-2016.

Firstly, 80 mL of 95 % ethanol is added to a clean conical flask, followed by the addition of 0.8 mL of phenolphthalein indicator. Then, the conical flask into is placed into 100 °C water bath heating until the ethanol slightly boiling. Once removed from the water bath, while the temperature of ethanol is still maintained above 70 °C, the ethanol is immediately titrated with the standard titration solution. When a slightly red color appears in the ethanol solution, and the color does not fade at least 15 seconds, the titration is stopped immediately, the acidity of the ethanol is neutralized. The neutralized ethanol solution is promptly poured into the sample, while it is still hot. And then the conical flask is returned to the water bath at 100 °C and heat until the ethanol boiling, after the conical flask is vigorously shaken to form a suspension. Finally, while it is still hot, the conical flask undergoes immediate titration using standard titration solution The end point of this second titration occurs when a slight

red color appears in the sample solution, and does not fade significantly within 15 seconds. At this point, and the number of standard titrimetric solution consumed is recorded as *V*. The hot ethanol indicator titration method does not require a blank test, the value of V_0 was zero.

$$X_{AV}(mg\ g-1) = \frac{(V - V_0) \times c \times 56.1}{m}$$

Where X_{AV} represents the acid value. V represents the volume of the standard titration solution consumed by the sample, measured in milliliter. V_0 represents the volume of the standard titration solution consumed by blank sample, also measured in milliliter. *c* represents the concentration of sodium thiosulfate standard solution, expressed in molar per liter. the molar mass of potassium hydroxide is 56.1 grams per molar. *m* represents the weight of grease, measured in gram.

2 Determination of POV

POV is determined by titrimetric method of the Chinese standard GB/T 5009.227-2016.

Petroleum ether is used to extract lipid from fish for testing. It is important to avoid conducting the lipid measurement under direct sunlight. 2g sample is added to 30 mL mixture of trichloromethane and glacial acetic acid, and gently shaking until the sample completely dissolve. Subsequently, 1.00mL saturated potassium iodide solution is added to the mixture, followed by plugged the cap tightly, and gently shaken for 30 seconds. Afterwards, the mixture is removed from darkness and supplemented with 100 mL water, which is then thoroughly shaken. Immediately sodium thiosulfate standard solution is used to titrate the precipitated iodine. If the estimated peroxide value exceeds 0.15 g $100g^{-1}$, a standard solution with the concentration of 0.01 mol L^{-1} should be used for titration. however, if the estimated peroxide value falls below this threshold, a standard solution with a concentration of 0.002 mol L^{-1} should be utilized instead. However, if the estimated peroxide value falls below this threshold, a standard solution with the concentration of 0.002 mol L^{-1} should be utilized instead. When the titration reaches a light yellow coloration point, 1 mL starch indicator should be added before continuing with further titration, and

vigorously shaking until the blue color of solution disappearing as the endpoint. Simultaneously, the blank test is carried out. The volume V of 0.01 mol L^{-1} sodium thiosulfate solution consumed by blank test is not more than 0.1mL.

When using the mass fraction of peroxide equivalent to iodine as peroxide value, the following formula is used for calculating.

$$X(g\ 100\ g-1) = \frac{(V - V_0) \times c \times 0.1269}{m} \times 100$$

Where X represents the peroxide value. V represents the volume of sodium thiosulfate standard solution consumed by sample, measured in milliliter. V_0 represents the volume of sodium thiosulfate standard solution consumed by the blank test, also measured in milliliter. c represents the concentration of sodium thiosulfate standard solution, expressed as molar per liter. 0.1269 represents mass of iodine equivalent to 1.00 milliliter of a standard titration solution of sodium thiosulfate, its concentration is 1.000 molar per liter. m represents the mass of the sample, its unit is grams. 100 represents the conversion factor. expressed as Finally, m stands for the mass of the sample being tested and is given in grams; this value is then multiplied by 100 to obtain a conversion factor.

3 Determination of TBARS

TBARS is determined according to the spectrophotometric method of the Chinese standard GB 5009.181-2016. Malondialdehyde, is an intermediate products of lipid oxidation, forms colored compounds with maximum absorption at 530nm when reacted with thiobarbituric acid (TBA).

5 g sample is added into a 100 mL stoppered conical flask, ensuring accuracy to the nearest hundredth, then accurately adding 50 mL trichloroacetic acid mixture, and vigorously shake well the solution. Subsequently, the sample is placed at 50 °C for 30 minutes, followed by cooling it to room temperature. Then filtered the solution using double-layer quantitative slow filter paper. The first filtrate is discarded, and the second filtrate is reserved. 5 mL above filtrate and standard series solution accurately is pipetted, and 5 mL trichloroacetic acid is taken as a blank sample, then adding 5 mL of aqueous thiobarbituric acid solution, mixing well, and the mixture is placed in a

90 °C water bath reaction for 30 minutes, then cooled to room temperature. Adjusting the zero point with a sample blank, the absorbance of the sample solution and the standard series solution is measured at 532 nm, and the standard curve is plotted with the mass concentration of the standard series solution on the horizontal coordinate, and the absorbance value on the vertical coordinate.

$$X(mg\ kg-1) = \frac{c \times V \times 1000}{m \times 1000}$$

where *X* represents the content of the sample. *c* denotes the concentration of malondialdehyde in the sample solution, calculated on the basis of the standard series curve, and expressed in microgram per milliliter. *V* represents the constant volume of the sample solution, measured in milliliter. *m* represents the sample mass, measured in gram. 1000 is the conversion coefficient of unit.
